# Classification of Space Objects by Using Deep Learning with Micro-Doppler Signature Images

**DOI:** 10.3390/s21134365

**Published:** 2021-06-25

**Authors:** Kwangyong Jung, Jae-In Lee, Nammoon Kim, Sunjin Oh, Dong-Wook Seo

**Affiliations:** 1 School of Electrical and Electronic Engineering, Yonsei University, Seoul 03722, Korea; kyjung@yonsei.ac.kr; 2 Interdisciplinary Major of Maritime AI Convergence, Korea Maritime & Ocean University (KMOU), Busan 49112, Korea; rhadodehfdl@gmail.com; 3 Department of Land Radar, Hanwha Systems, Yongin 17121, Korea; nammoon.kim@hanwha.com; 4 Agency for Defense Development, Daejeon 34075, Korea; sunjinoh@add.re.kr

**Keywords:** classification, convolution neural network, debris, deep learning, micro-doppler, space objects

## Abstract

Radar target classification is an important task in the missile defense system. State-of-the-art studies using micro-doppler frequency have been conducted to classify the space object targets. However, existing studies rely highly on feature extraction methods. Therefore, the generalization performance of the classifier is limited and there is room for improvement. Recently, to improve the classification performance, the popular approaches are to build a convolutional neural network (CNN) architecture with the help of transfer learning and use the generative adversarial network (GAN) to increase the training datasets. However, these methods still have drawbacks. First, they use only one feature to train the network. Therefore, the existing methods cannot guarantee that the classifier learns more robust target characteristics. Second, it is difficult to obtain large amounts of data that accurately mimic real-world target features by performing data augmentation via GAN instead of simulation. To mitigate the above problem, we propose a transfer learning-based parallel network with the spectrogram and the cadence velocity diagram (CVD) as the inputs. In addition, we obtain an EM simulation-based dataset. The radar-received signal is simulated according to a variety of dynamics using the concept of shooting and bouncing rays with relative aspect angles rather than the scattering center reconstruction method. Our proposed model is evaluated on our generated dataset. The proposed method achieved about 0.01 to 0.39% higher accuracy than the pre-trained networks with a single input feature.

## 1. Introduction

Ballistic missile (BM) classification is one of the most important problems for the ballistic missile defense (BMD) system. Among the three phases of flight of the target—boost, mid-course, and re-entry phases—the cone-shaped target flies for the longest period of time over the atmosphere during the mid-course phase [1]. In addition, during this phase, the cone-shaped targets are typically spin-stabilized so as not to deviate from their trajectory, and they exhibit precession and nutation motions under the effect of Earth’s gravity. Moreover, it is known that the precession rate is usually much slower than the spin rate and the precession angle is also relatively small compared to the half-cone angle [2]. By contrast, decoys and debris tumble due to the gravity, atmospheric resistance, and the absence of a spinning motor [3]. That is, the micro-motion of the cone-shaped targets is obviously different from that of decoys and debris [4]. The distinct micro-motions induce Doppler modulations on the returned radar signal known as the micro-Doppler frequency. For these reasons, many researchers have studied to classify the cone-shaped target and others by using the micro-Doppler frequency for several decades.

The procedure to classify the targets from the Doppler-modulated returned radar signal can be categorized into three steps: signature extraction, feature extraction, and classification. First, the time-varying micro-Doppler signature is usually obtained by applying the joint time-frequency (JTF) to the returned radar signal. The JTF analysis methods include the short-time Fourier transform (STFT), Wigner-Ville distribution, Choi-Williams distribution, Cohen’s class, and time-frequency distribution series (TFDS) [5]. Among these methods, the spectrogram is a commonly used method to display time-varying density of a time-varying signal. Recently, the cadence velocity diagram (CVD) [6], which is a Fourier transform of the spectrogram along the frequency bin, has also been used as a micro-Doppler signature.

Second, it is necessary to extract features from the micro-Doppler signatures in order for classifiers to classify the space objects. By combining the scattering center reconstruction (SCR) method with the STFT, the coning target micro-motion was extracted [4], [7]. By comparing the derived mathematic formulas with the STFT and Wigner-Ville distribution, the nutation and precession features were extracted [8,9,10]. Similarly, by using the derived mathematical formulas, the period and bandwidth of the micro-Doppler frequency were extracted [10]. In [11], the authors established the relationship between conning, spinning, and appearing frequencies of a cone-shaped target with empennages and extracted the features from the STFT by using the correlation figure method and additional fast Fourier transform (FFT). Single-range Doppler interferometry (SRDI)-based methods have also been introduced and used to extract micro-Doppler curves and to estimate feature parameters [12,13,14]. Besides, there are also attempts to extract features from the CVD. In [1], the authors described a three feature-extraction approach from CVD; they averaged and normalized the CVD (ACVD)-based, pseudo-Zernike-based, and Garbor-filter-based feature vector approaches and showed that all the three approaches have a good classification rate. I.O. Choi et al. introduced a simple 3D feature vector from the CVD [15]. There was a case in which feature vectors were extracted by applying the Radon transform to a high-resolution range profile frame in order to extract a 2-D target signature and calculating pZ moments from the 2-D target signature [3].

Finally, the extracted features were used as input data of various classifiers such as k-nearest neighbor classification, support vector machines, binary decision trees, and Naïve Bayes model [1,16]. However, the target classification performance of existing studies relied heavily on the feature extraction methods. Therefore, their generalization capability is limited and there is room for improvement.

In the series of procedures, the SCR method has been widely used to derive mathematic formulas and to simulate the returned radar signals. First of all, not only can the SCR method easily translate a mathematic dynamics formula into a mathematic micro-Doppler formula but also it can also be used directly to extract features from the STFT. Next, since the returned radar signal from a target can be expressed as the superposition of the signals from scattering centers, the SCR method makes it easy to simulate the returned radar signal. For more realistic returned radar signal, the effective scattering center and the occlusion effect were introduced [1,4,14]. More importantly, a lot of data is needed to train a classifier model, but it is practically difficult to gather measured, returned radar signals from real cone-shaped targets and debris with their distinct movements. Therefore, studies on the BM classification have mainly dealt with simulation results made from the SCR method. The SCR model not only can model all desired dynamic characteristics but also has a short calculation time, so this model makes it easy to calculate enormous volumes of data of the time-varying returned radar signal according to various dynamics. On the other hand, in the SCR method, the scattering coefficient of each scattering center, which is referred to as the point radar cross section (RCS) or scattering intensity, should be determined by analysis of the returned radar signal, which is obtained from measuring the radar signal or simulating the target model in an electromagnetic tool. In addition, the scattering coefficient is dependent on the target shape, material, frequency, incident, and scattered angles. Ironically, in order to simulate the radar signal, the real radar signal is necessary. For this reason, the SCR method has usually used the binary value, 0 or 1, as the scattering coefficient. The binary SCR model can simulate the time-varying micro-Doppler frequencies but cannot simulate the strength of each micro-Doppler frequency. Owing to the battle-field environment, the radar might be cluttered with noise, and the strength of the micro-Doppler needs to be simulated. Conventional approaches are not competent to train a robust classifier model in this circumstance.

Recently, deep learning techniques have been developed and widely used in radar target classification fields [16,17,18,19,20,21,22,23,24,25,26,27,28]. In particular, the convolutional neural network (CNN) can extract higher-level spatial features from lower-level layers via multiple convolutional layers, avoiding the manual feature extraction procedure of existing machine learning algorithms. In addition, the pooling and fully connected (FC) layers can solve the classification problem. Especially, in radar applications that use micro-Doppler images, CNN can extract local features and maintain velocity or frequency information on the extracted features [20]. Although CNN is widely used in radar image classification, research is underway to fully implement performance in the radar field due to the lack of training datasets.

Transfer learning is a supervised learning technique of reusing a pre-trained network with a much larger dataset. The main advantage is that the network has already learned many important features. Recent studies have been conducted with transfer learning based on the AlexNet [24], VGG-16 [26], and generative adversarial network (GAN) for classification of micro-Doppler images [21,22]. Similarly, pre-trained AlexNet and SqueezeNet have been used to classify the cone-shaped space target [23,27]. Using the RCS time series, the one-dimensional CNN structure has been proposed to classify the cone-shaped target and decoy [16]. Nevertheless, they all focus on feature extraction, data augmentation, and classification, and there is still room for improvement in studies that fuse extracted signatures such as spectrogram and CVD.

In this paper, we propose a parallel-structured CNN scheme that combines two types of micro-Doppler signatures: the spectrogram and CVD. By combining the time-varying density of the spectrogram with the local semantic characteristics of the CVD, we intend to improve classification performance. This idea starts with research showing that performance can be improved by simply fusing the features of different types of information with the same object, such as the camera image and depth information, the radar measurements, and lidar point cloud information. Thankfully, the spectrogram and CVD signatures we have obtained reveal different semantic representations of the same observed target. Since AlexNet has been introduced, efforts have continued to stack deep layers to improve classification performance. AlexNet has only five convolution layers, while VGG [26] and GoogleNet [28] have 19 and 22 layers, respectively. Recently, ResNet has been able to solve gradient vanishing problems and build deeper layers through the identity shortcut connection. Besides, the data set generated by using our inhouse shooting and bouncing rays (SBR) technique instead of the SCR model is used to train, validate, and test the proposed scheme. Our dataset reflects the intensity of the Doppler, enhancing the classifier’s performance even in noisy environments.

**Figure 1:**
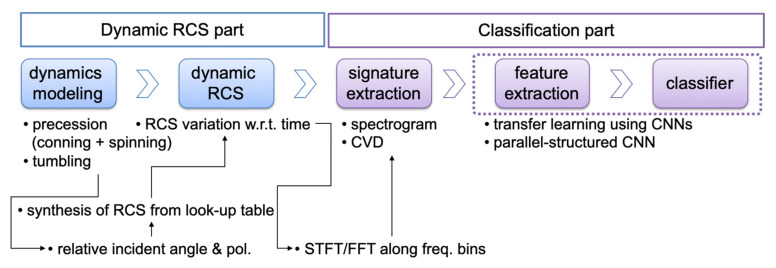
Flow diagram of our proposed work from estimating dynamic RCS to classifying targets.

Figure 1 shows the overall diagram of our proposed solution, which is organized with two parts: dynamic RCS pat and classification part. The dynamic RCS part includes the dynamic modeling of targets and estimating dynamic RCS of targets. In the classification part, the micro-Doppler signatures such as spectrogram and CVD are extracted from the dynamic RCS and the generated signature images are used to train and test the conventional and parallel-structed CNNs.

We use both useful information extracted from spectrogram and CVD using a parallelized structure. To analyze the impact of two signatures on classification performance, we adopt ResNet-18 with best classification performance in a single pre-trained network and implement parallel-structured CNN. The output feature maps of the two networks are then concatenated along the same dimension. The combined feature map is fed into the FC layer for global feature aggregation and classification.

The main contributions of this paper are as follows:

1. We propose a simple parallel-structured network that fuses spectrogram and CVD features based on the ResNet-18. Compared to a single pre-trained network backbone, the proposed method requires a shorter dwell time and achieves high classification accuracy.
2. We utilize the doppler strength that cannot be processed with SCR method in the data set. We believe that the EM simulation-based data augmentation method is a strong way to mimic a realistic and accurate data set. Based on our SBR [29] method, the proposed method can reflect fast dynamic RCS reflecting changes in the radar aspect angle.

The remainder of this paper is organized as follows. The models of cone-shaped targets and debris are introduced in Section 2. The deep learning network design of the proposed method is described in details in Section 3. Section 4 describes the experimental results and discussions of the proposed methods and comparisons with other works in the domain. Finally, Section 5 concludes the paper.

## 2. Models of Space Objects and Methods for Dynamic RCS Estimation

**Figure 2:**
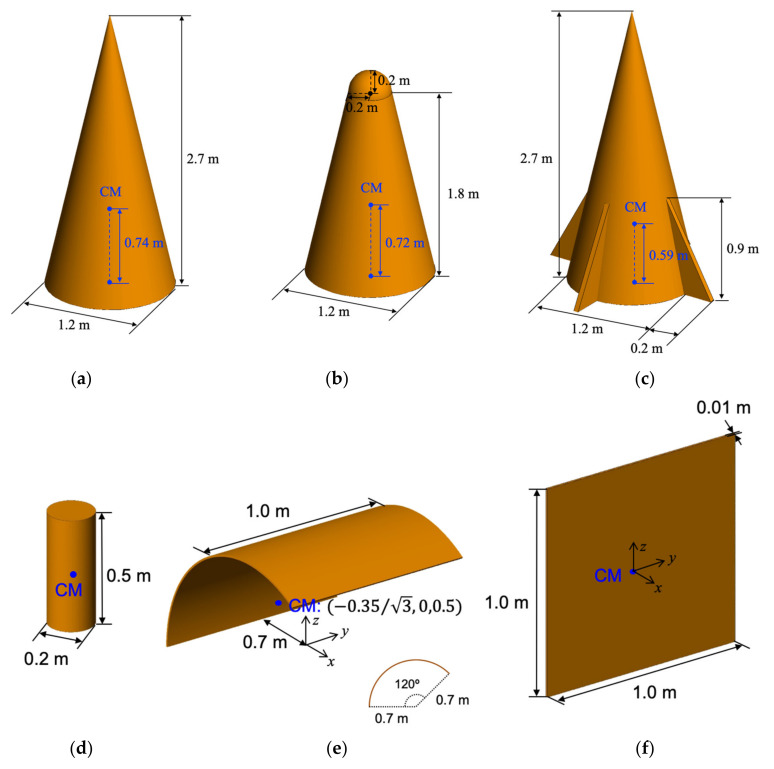
Space object model: (**a**) cone, (**b**) rounded cone, (**c**) cone with empennages, (**d**) cylinder, (**e**) curved plane, and (**f**) squared plane.

In this paper, three cone-shaped targets and three debris are considered: cone, rounded cone, cone with empennages, cylinder, curved plane, and squared plane, as shown in Figure 2. The cone-shaped target models are based on the same geometric cone and debris are considered in shapes that can be separated from missiles. The center of mass of each model is estimated by using the Altair Hypermesh.

**Figure 3:**
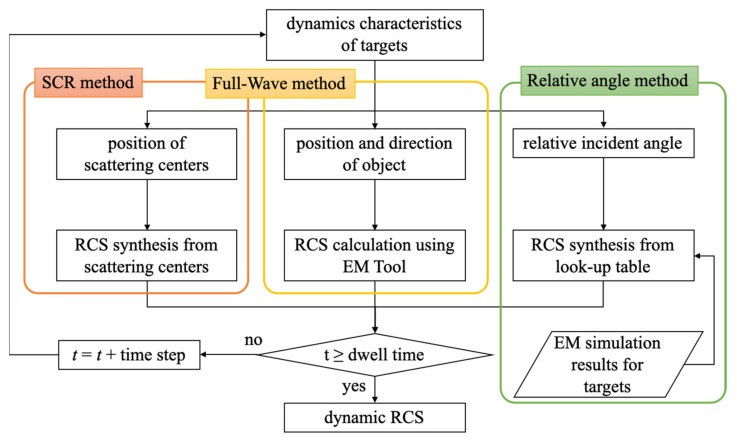
Flow charts of methods for dynamic RCS estimation.

We estimated the dynamic RCS, which is the squared magnitude of the scattered field with respect to time, under the assumption that the quasi-static mode is established on a frame-by-frame basis with sampling intervals instead of a time continuous model. As shown in Figure 3, the dynamic RCS can be obtained from three methods: SCR, full-wave, and relative angle methods. The pros and cons of these methods are summarized in Table 1. As mentioned before, the SCR method is a time-saving method, but it is only valid for simple targets. Contrastively, the full-wave method provides an accurate solution, but it is very time-consuming since the targets are rotated according to the dynamic characteristics at every time interval and are simulated by using an electromagnetic full-wave tool such as Altair’s FEKO and CST MWS. Meanwhile, the relative angle method utilizes a look-up table that configures the RCS values of a target with a fixed direction for all possible incident angles, and it uses the relative angle between the incident angle and the object direction as an input parameter of the look-up table instead of changing the target in real direction. It takes a lot of time to create the lookup table, but the relative angle method has a faster computation time than the full-wave method since it does not perform the electromagnetic analysis every time step like the full-wave method. Especially, the relative angle method has an advantage in the symmetric-structured target since the lookup table can be reduced. In our work, the relative angle method is used to calculate the dynamic RCS since the RCS of the cone-shaped targets does not vary with the azimuth angle, ϕ. Therefore, the lookup table is configured with the RCS values of cone-shaped targets with respect to the elevation angle. In practice, debris models that are not longitudinally symmetrical, as shown in Figure 2c,e,f, require a larger lookup table. The electromagnetic calculation of the targets was fulfilled by our in-house code, which is implemented by the SBR [29].

## 3. Dataset Generation

**Figure 4:**
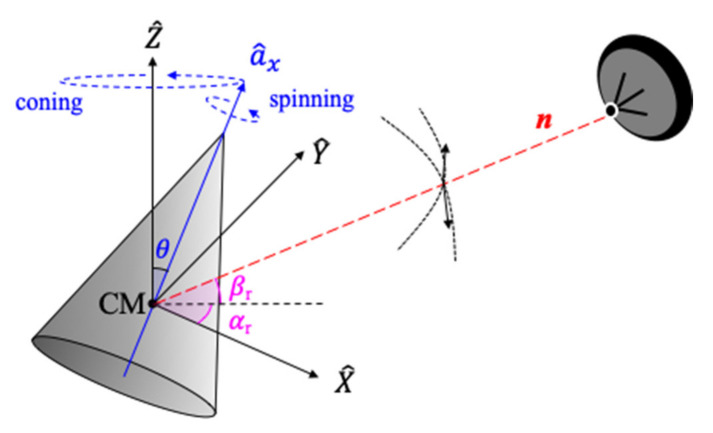
Geometry of radar and target with coning and spinning rotations.

As shown in Figure 4, we assume that the radar is stationary and the target’s center mass (CM) is located at the origin *O* of the reference coordinate system (*X*, *Y*, and *Z*). In order to generate the dataset for training and testing CNN models, first of all, the dynamics of targets should be defined. The dynamic parameters are summarized in Table 2, in which the values are based on the actual dynamics of cone-shaped targets and debris [3]. The space objects have the precession and tumbling motions, respectively, with a rotation rate of 0.25–3 Hz. The precession angle (θ) of the cone-shaped target is set in the range of 4 degrees to 12 degrees, while tumbling debris have a precession angle of 90 degrees. In addition, the precession axis of cone-shaped target points is in the direction of travel, while debris tumble is in random direction. Therefore, the initial direction of debris is random in the range of 0 degree to 180 degrees. The incident angle (β_r_) is defined as the angle between the radar line of sight (RLOS, ***n***) and normal plane of the target trajectory, and it ranges from 0 degree to 85 degrees in order to cover the mid-course phase. Since it is difficult to simulate all the value of the parameter range, the parameters are changed by their step in Table 2 to simulate the dynamics of targets.

Next, to simulate the radar signal returned from a target, the radar parameters are required. Assume that the radar operates at a X-band; that the pulse repetition frequency (PRF) is 5 kHz; and that these pulses are transmitted during the dwell times of 1, 2, and 4 s. These parameters are summarized in Table 3 and are chosen by referring previous literatures [6,15,16,30,31]. Especially, it is known that the detection of the micro-Doppler modulation generated by vibration may not be possible at low radar frequency bands, and it is recommended that the operating frequency should be the X-band (8–12 GHz) for detecting centimeter-level displacement [30].

**Figure 5:**
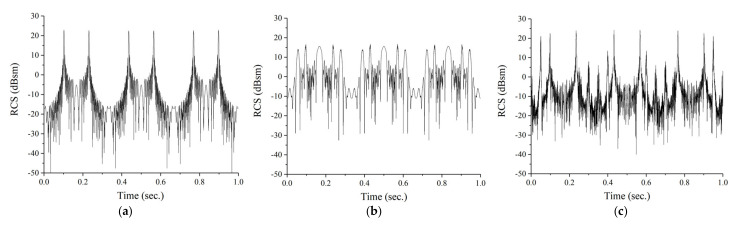
Estimated dynamic RCS during dwell time of 1 s for (**a**) cone, (**b**) rounded cone, and (**c**) cone with empennages.

Figure 5 shows the estimated RCS versus time curve of the cone-shaped target models with a rotation rate of 3 Hz, a precession angle of 10 degrees, and an incident angle of 5 degrees. For the cone model, the dynamic RCS has peaks when the incident field normally illuminates the cone’s side surface. For the dynamic RCS of the rounded cone, since the rounded tip strongly reflects the incident field, there are more peak points. Meanwhile, the cone with empennages has more complex RCS pattern due to empennages causing multi-path. In these figures, we can roughly notice that the rotation rate is about 3 Hz and the dynamic RCS has different patterns depending on the shape of cone-shaped target.

**Figure 6:**
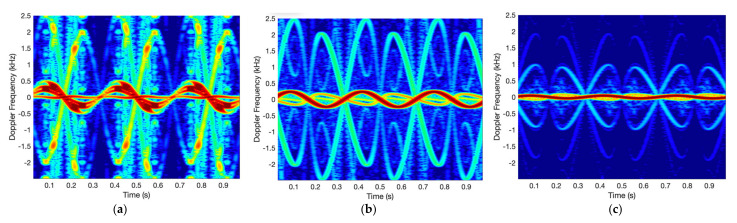
Spectrogram of rounded cone for (**a**) β_r_ = 0°, (**b**) 60°, and (**c**) 85°.

Since we use CNNs to classify the cone-shaped target and debris, radar signatures in the form of 2-dimensional (2D) image such as the spectrogram and CVD are required. To obtain the spectrogram, in our simulation, the Kaiser window, 512 DFT points, and an overlap of 255 are used. Figure 6 shows the spectrogram of the rounded cone for the incident angles (β_r_) of 0, 30, 60, and 85 degrees when the rotation rate is 3 Hz. For β_r_ = 0°, the micro-Doppler signal by the tip appears with a large amplitude in red, and those by other scattering points appear with smaller amplitudes. β_r_ = 85°, that is, the radar is located in front of the cone-shaped target tip, and the micro-motions of the cone-shaped target are almost on the normal plane of the RLOS. Therefore, as the incident angle increases, the variation of the micro-Doppler frequency becomes smaller. On the other hand, the yellow and green lines appear as a result of Doppler aliasing [5].

**Figure 7:**
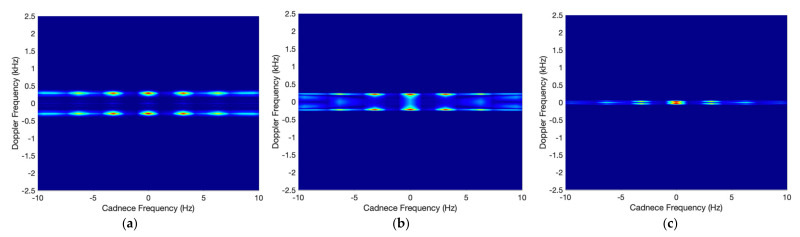
CVD of rounded cone for (**a**) β_r_ = 0°, (**b**) 60°, and (**c**) 85°.

**Figure 8:**
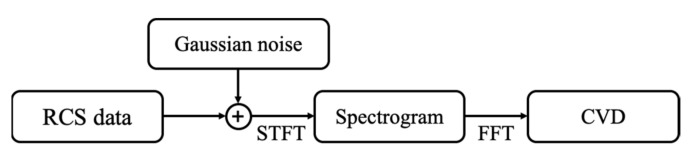
Diagram for calculating spectrogram and CVD from RCS data.

The CVD is commonly used as a measure on how often the difference velocities repeat (‘cadence frequencies’). The CVD is obtained by Fourier transform of spectrogram along frequency bins. Therefore, we obtain the spectrogram from a radar signal and the CVD from the spectrogram. Figure 7 shows the CVDs corresponding to the spectrogram of Figure 8. As seen in the figures, the first peak of the cadence frequency is at about 3 Hz that means the rotation rate. In addition, for β_r_ = 0°, the micro-Doppler frequency by the tip appears near 0.3 Hz. As confirmed by the Spectrogram results, the maximum micro-Doppler frequency also decreases as the incident angle increases.

In the radar signal calculated earlier, no noise was taken into account. In practice, there are many noise sources at the radar receiver. In addition to noise generated by the radar receiver, background noise collected from the antenna appears at the radar receiver. To consider the noise effect, the white Gaussian noise is added to the radar signal with the SNR varying by 5 dB in the range of −10 to 20 dB, as shown in Figure 8.

**Figure 9:**
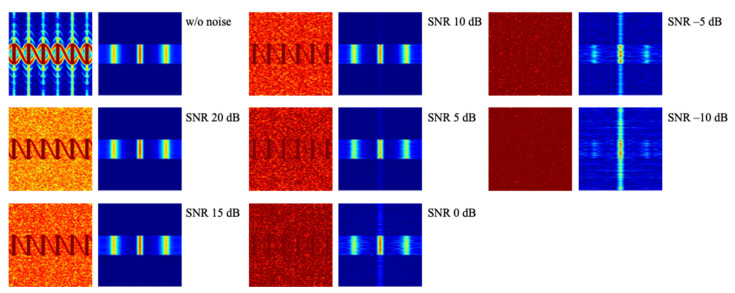
Spectrogram and CVD by tumbling motion of plate model with respect to several SNRs.

Figure 9 shows examples of spectrogram and CVD for the plate debris model with respect to the noise level. As the SNR is lower, the background noise level of the spectrogram is higher and represented by red color, while the CVD has a stronger blur effect. Since the CVD is a Fourier transform of the spectrogram along frequency bin, noise components without periodic characteristics in the spectrogram can be reduced through the FFT and appear as a blur effect in the CVD.

Table 4 shows the number of generated micro-Doppler signature images with and without noise for each target, dwell time, and signature type. The cone-shaped target models have a wide precession angle, while debris models have a more diverse initial direction. Therefore, the number of signature images for debris model is larger than that for cone-shaped target models.

## 4. Deep Neural Network and Classification Results

For the baseline, CNN transfer learning is used to classify the space objects using the micro-Doppler signatures of the spectrogram and CVD in a supervised manner. The signature images used in this work have corresponding labels identifying the model names. The pre-trained CNNs are the AlexNet, VGG-19, GoogLeNet, ResNet-18, and ResNet-50. The fine-tuning, re-training, and testing are performed by using a MATLAB on a personal computer with intel i7-9700 8 cores, 128 GB RAM, and GeForce Titan X with 12 GB memory. The generated dataset is randomly divided into three subsets: the training set, the validation set, and the test set. The ratios used are: 80% train, 10% validate, and 10% test. The sgdm (stochastic gradient descent with momentum) is used as the solver for training network, and we use the learning rate of 0.001.

Table 5 summarizes the performance evaluation of the transfer learned CNNs using the spectrogram and CVD of the micro-Doppler signal for space objects in a noise environment. The ResNet-18 has good classification accuracy for spectrogram and CVD, as well as a relatively short training period. The training time of VGG-19 takes about six times longer than ResNet-18, but its classification accuracy is not as good as ResNet-18. Interestingly, ResNet-18 has better performance than ResNet-50, which has a deeper structure than ResNet-18. This may be because that the training data is not sufficient to train the ResNet-50 with deep layers.

The maximum and minimum classification accuracies of the single networks for spectrogram and CVD are shown in Table 6. The transfer-learned CNN models have an accuracy of more than about 95% for spectrogram, while they have an accuracy of less than about 92% for CVD.

**Figure 10:**
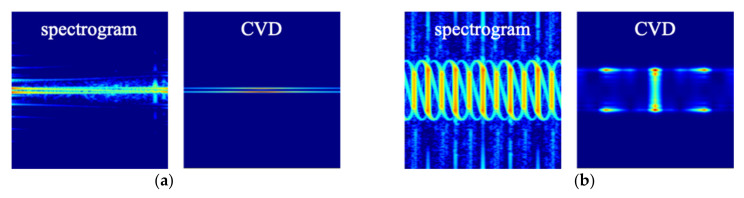
Spectrogram and CVD of curved plate model with the rotation rates of (**a**) 0.25 Hz and (**b**) 3 Hz.

Spectrogram has various patterns according to dynamic parameter and models, while CVD has relatively simple patterns, as shown in Figure 9. Considering the characteristics of the CNNs that extract features based on the pattern of the image, the spectrogram is more suitable for classification using CNN than CVD. On the other hand, as the dwell time increases, the results learned by spectrogram and CVD have higher accuracy. The micro-Doppler signatures are generated for the target with the rotation rate of 0.25 to 3 Hz. If the rotation rate is 0.25 Hz, only 1/4 cycle of the signal is received during the dwell time of 1 s. Moreover, the low rotation rate has small micro-Doppler frequency, as shown in Figure 10. Therefore, the short dwell time and low rotation rate reduce the accuracy.

**Figure 11:**
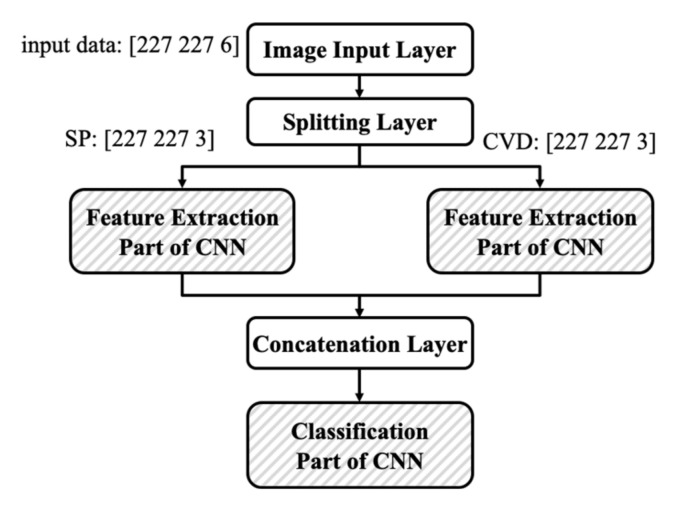
Structure of the parallel-structured ResNet-18 for two types of input image.

For higher accuracy, we construct two identical CNNs in parallel in which one CNN has the spectrogram and the other CNN has the CVD as the input image, as shown in Figure 11. Since the network generally is a combination of two components: feature extraction part (convolution and pooling layers) and the classification part (fully connected layers), two identical feature extraction parts are arranged in parallel and the outputs of two parts are concatenated and used as an input of one classification part. According to the results of Table 4, the ResNet-18 has high accuracy as well as short training time. Therefore, the ResNet-18 is used to construct the parallel-structured CNN model.

The test performance of the parallel-structured CNN model is summarized in the last column of Table 5. It can be seen that the parallel-structured CNN has higher accuracy than the single CNN architecture models. In the case of VGG19, the results of using spectrogram exhibited the best performance in Dwell time of 1 s, but the training time took the longest. The proposed parallel-structured ResNet-18 model improves accuracy of about 0.01% to 0.39% compared to the maximum classification accuracy of the ResNet-18 in Table 5. In addition, since the two networks trained with spectrogram and CVD, the training time only increases slightly.

## 5. Conclusions

This paper proposed the parallel-structured CNN model for classifying the space objects. To train the CNN model, the dynamics of the cone-shaped target and debris are simulated and the radar signals by the dynamics are obtained by the relative concept. Then, the radar signal is transformed into the micro-Doppler signatures of the spectrogram and CVD by the STFT and FFT, respectively. A bunch of the 2D signature images is collected as the dataset that is used to transfer-learn the well-known CNNs. The transfer-learned CNNs by the spectrogram have accuracy of more than 95%, while those learned by the CVD have relatively low (about 92%) accuracy. Compared to these results, the proposed parallel-structured ResNet-18 has high accuracy of more than 97%. As a future work, we want to create a large database with many images and various real space object target models. In addition, we are planning to design a network architecture that is lightweight and considers the association between the two images for actual equipment application.

## Figures and Tables

**Table 1:** Dynamic parameters for dataset generation.

Methods	Computation Time	Accuracy	Etc.
SCR method	Short	Poor	Simple model only
Full-wave method	Long	High	All model available
Relative angle method	Medium	High	Suitable for symmetrical structure

**Table 2:** Dynamic parameters for dataset generation.

Parameter	Values	Step
Rotation rate	0.25–3 Hz	0.25 Hz
Incident angle (β_r_):	0–85°	5°
Precession angle (θ)	4–12°	2°
Initial direction of debris (θ_init_)	0–180°	10°

**Table 3:** Radar system parameters.

Parameter	Values	Etc.
Carrier frequency	X-band	
Sampling frequency	5 kHz	Pulse repetition frequency Time step: 0.2 ms
Dwell time	1, 2, and 4 s	

**Table 4:** Number of generated micro-Doppler signature images.

Dwell Time	Signature	Cone	Rounded Cone	Cone w/ Empennages	Cylinder	Curved Plane	Squared Plane	Total
1 s	SP ^1^	8640	8640	8640	1728	32,832	32,832	93,312
	CVD	8640	8640	8640	1728	32,832	32,832	93,312
2 s	SP	8640	8640	8640	1728	32,832	32,832	93,312
	CVD	8640	8640	8640	1728	32,832	32,832	93,312
4 s	SP	8640	8640	8640	1728	32,832	32,832	93,312
	CVD	8640	8640	8640	1728	32,832	32,832	93,312

^1^ SP: spectrogram.

**Table 5:** Accuracy of conventional transfer learned CNNs and parallel-structured ResNet-18 (Ours).

Dwell Time	AlexNet		VGG19		GoogLeNet		ResNet-18		ResNet-50		Ours
	SP	CVD	SP	CVD	SP	CVD	SP	CVD	SP	CVD	Parallel
1 s	95.83%	88.02%	97.52%	87.56%	96.32%	87.65%	97.25%	89.65%	96.88%	87.84%	97.26%
2 s	97.00%	88.41%	98.03%	88.63%	97.25%	89.33%	98.06%	91.03%	97.50%	90.01%	98.22%
4 s	97.02%	91.02%	98.39%	91.08%	97.20%	89.15%	98.40%	91.79%	97.66%	91.41%	98.79%
training time	~60 min		~480 min		~90 min		~76 min		~180 min		~100 min

**Table 6:** Minimum and maximum accuracies of the single networks.

Dwell Time	Spectogram		CVD	
	Min.	Max.	Min.	Max.
1 s	95.83%	97.52%	88.02%	89.65%
2 s	97.00%	98.06%	88.41%	91.03%
4 s	97.02%	98.40%	91.02%	91.79%

## Data Availability

Not applicable.
